# Comparison of the protective effect of cytosolic and mitochondrial Peroxiredoxin 5 against glutamate-induced neuronal cell death

**DOI:** 10.1080/13510002.2021.1901028

**Published:** 2021-03-15

**Authors:** Mi Hye Kim, Da Yeon Kim, Hong Jun Lee, Young-Ho Park, Jae-Won Huh, Dong-Seok Lee

**Affiliations:** a School of Life sciences, BK21 Plus KNU Creative BioResearch Group, Kyungpook National University, Daegu, Republic of Korea; b College of Natural Sciences, Kyungpook National University, Daegu, Republic of Korea; c Department of Physiology, Stem Cell Research Center, Pusan National University School of Medicine, Yangsan, Republic of Korea; d College of Medicine, Chungbuk National University, Cheongju, Republic of Korea; e Research Institute, e-biogen Inc., Seoul, Republic of Korea; f Futuristic Animal Resource and Research Center, Korea Research Institute of Bioscience and Biotechnology (KRIBB), Cheongju, Republic of Korea; g National Primate Research Center, Korea Research Institute of Bioscience and Biotechnology (KRIBB), Cheongju, Republic of Korea

**Keywords:** Cytosolic ROS, mitochondrial ROS, apoptosis, Peroxiredoxin 5, glutamate, HT22, Hyper, Hydrogen peroxide

## Abstract

**Objectives**: Although glutamate is an essential factor in the neuronal system, excess glutamate can produce excitotoxicity. We previously reported that Peroxiredoxin 5 (Prx5) protects neuronal cells from glutamate toxicity via its antioxidant effects. However, it is unclear whether cytosolic or mitochondrial Prx5 provides greater neuroprotection. Here, we investigated differences in the neuroprotective effects of cytosolic and mitochondrial Prx5.

**Methods**: We analyzed patterns of cytosolic and mitochondrial H_2_O_2_ generation in glutamate toxicity using HyPer protein. And then, we confirmed the change of intracellular ROS level and apoptosis with respective methods. The mitochondrial dynamics was assessed with confocal microscope imaging and western blotting.

**Results**: We found that the level of mitochondrial H_2_O_2_ greatly increased compared to cytosolic H_2_O_2_ and it affected cytosolic H_2_O_2_ generation after glutamate treatment. In addition, we confirmed that mitochondrial Prx5 provides more effective neuroprotection than cytosolic Prx5.

**Discussion**: Overall, our study reveals the mechanisms of cytosolic and mitochondrial ROS in glutamate toxicity. Our findings suggest that mitochondrial ROS and Prx5 are attractive therapeutic targets and that controlling these factors be useful for the prevention of neurodegenerative diseases.

## 1. Introduction

Reactive oxygen species (ROS), such as superoxide anion (O_2_^-^), hydrogen peroxide (H_2_O_2_), and hydroxyl radical (OH), are chemically active molecules [1] that participate in the regulation of biological and physiological processes in cellular systems, for example, by serving as initiators of signal transduction in several pathways [2]. However, high levels of ROS can cause damage to DNA, proteins, and lipids, leading to negative effects on cellular homeostasis. Consequently, ROS may be important factors in several pathological conditions [3]. The main sources of ROS are mitochondria and NADPH oxidases (NOXs). Unlike NOX-generated ROS, ROS derived from mitochondria are regarded as byproducts of oxidative metabolism; they are produced primarily via electron transfer in the mitochondrial respiratory chain [4]. A growing body of evidence has shown that mitochondria and mitochondrial ROS are linked to several chronic diseases, including neurodegenerative diseases [5,6].

A contributing factor in many neurodegenerative conditions is excessive glutamate release, which leads to excitotoxicity and excessive ROS generation, resulting in neuronal damage [7]. Glutamate-induced neuronal cell death is considered to be a cause of neurodegenerative disease [8,9]. In glutamate toxicity, high levels of ROS accelerate apoptotic cell death through the breakdown of intracellular Ca^2+^ homeostasis, mitochondrial dysfunction, and overproduction of ROS [10]. Numerous studies have reported that modulating ROS levels using antioxidant enzymes, therefore, constitutes an appropriate strategy for the improvement of glutamate toxicity [11,12]. In our previous study, we demonstrated that Peroxiredoxin 5 (Prx5), which is a potent antioxidant enzyme, helps to protect against neuronal cell death induced by glutamate toxicity. We focused particularly on the correlation between glutamate-induced apoptosis and cytosolic/mitochondrial ROS. Our findings suggested a need for research on the casual relationship between cytosolic and mitochondrial ROS in glutamate-induced apoptosis [13]. It is not yet known whether cytosolic or mitochondrial ROS is of greater importance in glutamate toxicity.

Prx5, a mammalian thioredoxin peroxidase, is intracellularly localized to mitochondria, cytosol, peroxisomes, and nuclei [14]. The distributed subcellular localization of Prx5 compared to other peroxiredoxins implies the essential function of Prx5 in the cellular system. In particular, since Prx5 is located mainly within cytosol and mitochondria, which are known sources of ROS, Prx5 is thought to have extensive antioxidant effects [15]. In the present study, we focused on the specific roles of Prx5 in these two different subcellular compartments (cytosol and mitochondria). In addition, we explored the tendency of cytosolic and mitochondrial ROS (in priority H_2_O_2_) generation in glutamate toxicity using lentivector-mediated HT22 cells expressing HyPer, an H_2_O_2_-sensitive fluorescence indicator. In this way, the present study demonstrates the protective effect of cytosolic Prx5 (CytPrx5) and mitochondrial Prx5 (MtPrx5) against glutamate toxicity. Furthermore, we identified the specific subcellular origin of ROS, which has a direct influence on glutamate-induced neuronal cell death.

## 2. Materials and methods

### 2.1. Chemicals and reagents

Glutamate was purchased from Sigma-Aldrich (MO, USA). Dulbecco’s modified Eagle’s medium (DMEM) and penicillin/streptomycin were purchased from Welgene (Daegu, Korea).

### 2.2. Cell culture and glutamate treatment

Mouse hippocampal neuronal HT22 cells were cultured in 5% CO_2_ and at 37°C in DMEM containing 4500 mg/L glucose and supplemented with 1% penicillin/streptomycin and 10% fetal bovine serum (FBS; Gibco, New Zealand). For glutamate treatment, the cells were cultured in 6-well plates (SPL Life Sciences Co., Pocheon-si, Korea), then, 12 h later, incubated with 5 mM glutamate (Sigma-Aldrich, MO, USA) for 12 h.

### 2.3. Plasmid cloning and establishment of stable cell lines

The HyPer-Cyto gene was obtained from pHyPer-Cyto (Evrogen, Russia, Moscow), and the HyPer-Mito gene was generated by fuzing the mitochondrial targeting sequence (MTS) with the HyPer-Cyto gene. The HyPer-Cyto and HyPer-Mito genes were inserted into a pLenti6.3/V5-DEST vector (Invitrogen, CA, USA). The Prx5 gene, provided by Dr. Tae-Hoon Lee (Chonnam National University, Gwangju, Korea), was cloned to generate recombinant plasmids. MtPrx5, which contains the MTS region on its N-terminal region, was generated by removing the stop codon from Prx5 and amplifying the MTS by PCR using LA Taq polymerase (Takara, Shiga, Japan), then cloning the final gene into a pCR8/GW/TOPO vector (Invitrogen). For the construction of CytPrx5, both the MTS region and stop codon of Prx5 were eliminated. Then, the CytPrx5 and MtPrx5 genes were inserted into the pLenti6.3/V5-DEST vector using LR clonase (Invitrogen). To establish a stable cell line, HT22 cells were seeded in 6-well plates. After 24 h, the cells were transfected using Effectene (Qiagen, CA, USA) according to the manufacturer's instructions. After 24 h, the transfected cells were selected using 8 μg/mL blasticidin (Invitrogen) for 7 d.

### 2.4. Analysis of cell viability

Cell viability was measured by 3-(4,5-dimethyl-2-thiazolyl)-2,5-diphenyl-2H-tetrazolium bromide (MTT) assay; HT22 cells (5 × 10^3^ cells of each type) were cultured in 6-well plates for 12 h before treatment with glutamate. The cells were then incubated with 5 mM glutamate for 12 h. Then, the culture medium was carefully removed and replaced with 0.5 mg/mL MTT solution dissolved in phenol red–free DMEM. The cells were then incubated for 1 h at 37°C. Next, the medium was removed and 500 μL DMSO was added to each well to dissolve the formazan crystals. Absorbance was measured at 550 nm with an Infinite F50 microplate reader (TECAN, Männedorf, Switzerland).

### 2.5. Annexin V and propidium iodide staining

An Annexin V-FITC/Propidium Iodide (PI) Apoptosis Detection Kit (BD Biosciences, CA, USA) was used to detect apoptosis by flow cytometry. Staining was conducted according to the manufacturer’s instructions. After glutamate treatment, cells were collected and washed with phosphate-buffered saline (PBS). Next, annexin V (5 μL) and PI (5 μL) were added to the cell suspensions, and cells were incubated for 10 min at 37°C in the dark. Then, the cells were analyzed by flow cytometry.

### 2.6. Detection of intracellular ROS and calcium level

Intracellular ROS were detected using 2′,7′-dichlorofluorescein diacetate (CM-H_2_DCF-DA; Invitrogen). Intracellular calcium levels were measured using Fluo-4 AM (Thermo Fisher Scientific). First, HT22, HT22-CytPrx5, and HT22-MtPrx5 cells were seeded in 6-well plates. Following 12 h of incubation, they were treated with 5 mM glutamate for 12 h. Control cells were not treated. Then, cells were harvested using 0.05% trypsin-EDTA. The collected cells were washed with PBS (pH 7.4) and incubated with 2.5 μM DCF-DA and Fluo-4 AM for 20 min at 37°C. Then, the cells were washed twice with PBS and analyzed by flow cytometry (BD Biosciences). The level of H_2_O_2_ was measured by using Fluorimetric Hydrogen Peroxide Assay Kit (Sigma-Aldrich) following the protocols provided by manufacturer. The fluorescence was measured by a microplate reader at 540/590 nm.

### 2.7. Protein extraction and western blot analysis

Whole protein lysates were extracted from cells using PRO-PREP Protein Extraction Solution (Intron Biotechnology, Seongnam, Korea). Cytoplasmic and mitochondrial fractionation was performed using a mitochondrial isolation kit for cultured cell (Thermo Scientific, MA, USA) according to the manufacturer’s protocol. Proteins were separated by 8–12% SDS-PAGE and then transferred onto nitrocellulose membranes (Pall, FL, USA). Membranes were incubated with antibodies against dynamin-related protein 1 (Drp1) (Cat#sc-32898), Mfn1 (Cat#sc-50330), Mfn2 (Cat#sc-50331) (Santa Cruz, CA, USA), p-Drp1 (Ser637) (Cat#4897S), cleaved caspase-3 (Cat#9661s), calcineurin (Cat#2614S; Cell Signaling, Danvers, MA, USA), Prx5 (Cat#LF-PA0210), and β-actin (Cat#LF-PA0207) (Abfrontier, Korea). We used horseradish peroxidase-conjugated anti-mouse and anti-rabbit IgGs (Thermo Scientific) as secondary antibodies. Protein bands were visualized with Clarity Western ECL Substrate (Bio-Rad, CA, USA), and band intensities were analyzed with Multi Gauge version 3.0 software (Fujifilm, Japan).

### 2.8. Mitochondrial imaging and analysis

For mitochondrial analysis, HT22 cells were seeded on poly-D-lysine-coated glass. The cells were then treated with glutamate for 12 h. Subsequently, the cells were stained with MitoTracker Red CMXRos (Thermo Scientific) and fixed with 4% paraformaldehyde (Sigma-Aldrich). Images of the cells were acquired with an LSM-710 confocal microscope (Carl Zeiss, Jena, Germany), and the mitochondrial length was measured with ImageJ software (NIH, Bethesda, MD, USA). The mitochondrial length was randomly measured, as previously described [16], from more than 50 mitochondrial particles per cell in over 20 cells.

### 2.9. Immunocytochemistry

HT22 and CytPrx5-/MtPrx5-V5 transfected HT22 cells were seeded on to poly-D-lysine-coated six-well plates. Cells were fixed with 4% paraformaldehyde (Sigma-Aldrich) and blocked with 1% BSA in PBST for 30 min. V5 antibody in blocking solution was added, and cells were incubated overnight. The following day, cells were treated with secondary antibody and Alexa Fluor 488 (Thermo Scientific). And then, three types of HT22 cells were stained with DAPI or Mitotracker Red CMXRos fluorescent dye. Images were acquired using an LSM-710 confocal microscope (Carl Zeiss, Jena, Germany).

### 2.10. Statistical analysis

All experiments were repeated at least three times. Quantitative data are presented as the mean ± SD of replicates. Data were analyzed using analysis of variance on GraphPad Prism 5.01 software (GraphPad Software Incorporated, CA, USA). *P* values lower than 0.05 were considered statistically significant.

## 3. Results

### 3.1. Glutamate upregulated the expression level of CytPrx5 and MtPrx5

**Figure 1.**
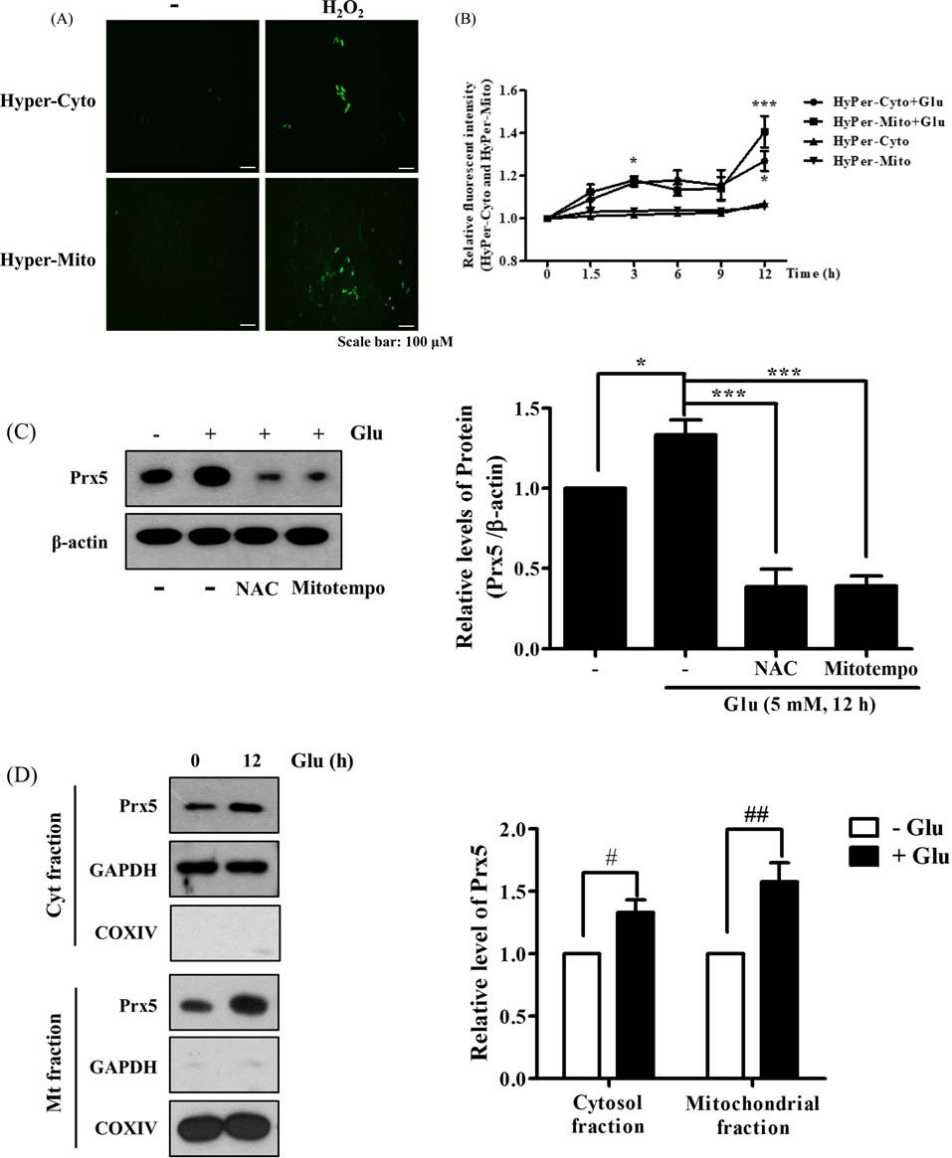
Changes in cytosolic and mitochondrial ROS levels and Prx5 expression in glutamate-treated HT22 cells. (A) The fluorescence intensity of HyPer-cyto and HyPer-mito to HT22 cell treated with H_2_O_2_ (100 μM) for 30 min. (B) HT22 cells expressing HyPer-Cyto and HyPer-Mito treated with 5 mM glutamate for the indicated times (0, 1.5, 3, 6, 9 and 12 h) were assessed by flow cytometry. (C) Western blot analysis was used to measure the expression of Prx5. HT22 cells were treated with glutamate for 12 h following treated with the 10 mM N-acetyl-L-cysteine (NAC) or 200 μM MitoTEMPO for 30 min. HT22 cells treated with NAC or MitoTEMPO were used as positive control. (D) Protein expression of Prx5 in cytosolic and mitochondrial fractions was confirmed by western blotting in HT22 cells treated with 5 mM glutamate for 12 h. Cytoplasmic and Mitochondrial fractionation were performed using a mitochondrial isolation kit. Values represent the mean ± SD (*n* = 4). *P < 0.05; **P < 0.01; ***P < 0.001 vs. glutamate treated HT22 cells. #*P* < 0.05; ##*P* < 0.01 vs. glutamate untreated HT22 cells.

Glutamate is known to produce cytosolic and mitochondrial ROS, resulting in stimulation of intracellular antioxidant systems [17]. To figure out the pattern of H_2_O_2_ generation by glutamate treatment, we designed HT22 cells that stably expressed HyPer protein specifically in the cytosol and mitochondria (HyPer-Cyto and HyPer-Mito, respectively). Then, we confirmed the sensitivity of HyPer-Cyto and HyPer-Mito to H_2_O_2_. As a result, H_2_O_2_ treatment increased the fluorescence intensity (Figure 1(A)). Next, we identified time-dependent changes in HyPer fluorescence intensity after glutamate treatment using flow cytometry. As shown in Figure 1(B), glutamate increased HyPer-Cyto and HyPer-Mito fluorescence to similar levels for up to 9 h after treatment. However, HyPer-Mito showed a dramatic increase compared to HyPer-Cyto at 12 h. In a previous study, we confirmed that glutamate treatment increased the expression level of Prx5 in a time-/concentration-dependent manner and that glutamate-induced ROS generation was related to the upregulation of Prx5 expression [13]. The present study showed that co-treatment with N-acetyl-L-cysteine (an intensive ROS scavenger) or MitoTEMPO (a mitochondrial ROS scavenger) downregulated Prx5 expression, indicating that the upregulation of Prx5 was related to glutamate-induced ROS generation (Figure 1(C)). These results are corresponded to previous study [18]. Since Prx5 is known to be broadly dispersed inside cells, including in the cytosol and mitochondria [19], we confirmed the subcellular distribution of Prx5 induced by glutamate using cytosolic and mitochondrial cell fractions. We found that the level of Prx5 increased in both cytosol and mitochondria, but MtPrx5 was upregulated to a much greater degree than CytPrx5 (Figure 1(D)). The above results imply that both cytosolic and mitochondrial H_2_O_2_ are generated by glutamate toxicity.

### 3.2. Generation of cell lines stably expressing CytPrx5/MtPrx5

**Figure 2.**
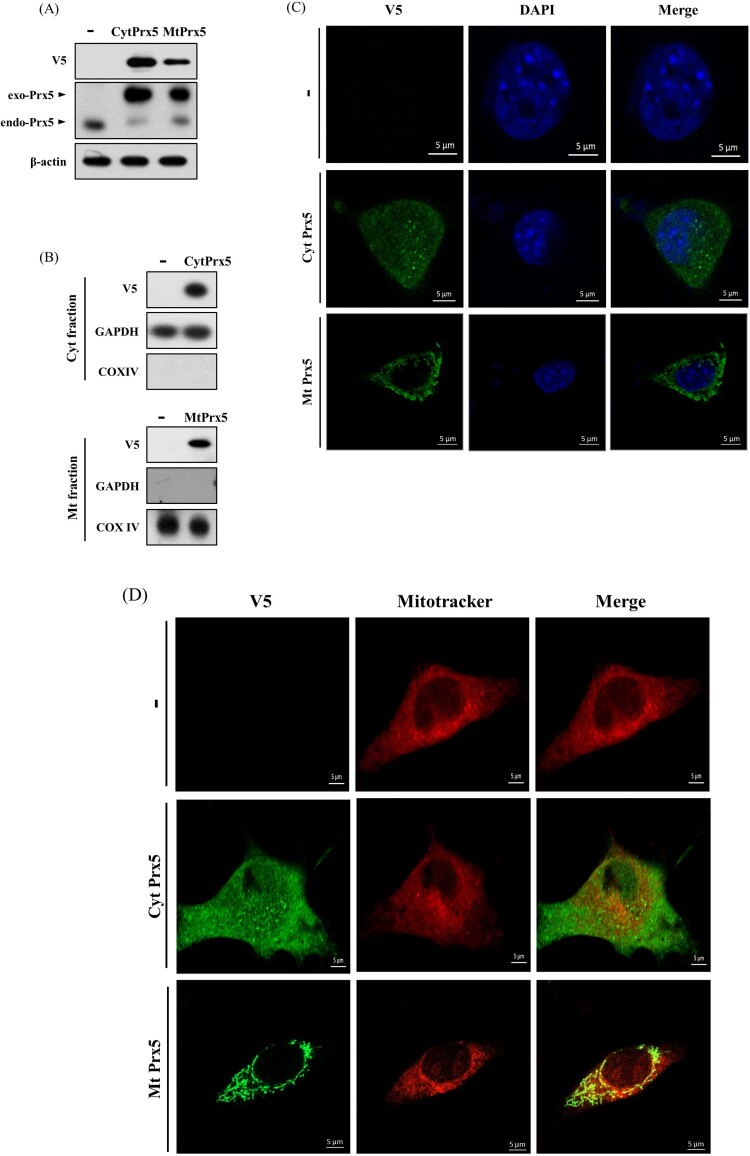
Confirmation of subcellular localization of Prx5 in HT22 cells transfected with targeted Prx5. (A) Stable expression of exogenous Prx5 targeted to cytosol or mitochondria was detected by western blotting with Prx5 and V5-tag antibodies. (B) Cytosolic or mitochondrial localization of Prx5 was confirmed by western blotting. Cytoplasmic and Mitochondrial fractionation were performed using a mitochondrial isolation kit. (C and D) The localization of cytosolic/mitochondrial Prx5 was detected by immunocytochemical analysis. Three types of HT22 cells were stained with DAPI (blue) or 1 μM Mitotracker (red) fluorescent dye after carrying out immunocytochemistry with V5-tag antibody (green). exo-Prx5; exogenous Prx5, endo-Prx5; endogenous Prx5.

To evaluate the protective effect of CytPrx5 and MtPrx5 against glutamate toxicity, we established a stable cell line in which Prx5 is expressed only in cytosol or mitochondria. CytPrx5 was produced by removing mitochondrial targeting sequence (the MTS from Prx5), and MtPrx5 was produced by amplifying the MTS. To begin, we confirmed the exogenous expression of CytPrx5 and MtPrx5 by western blotting using Prx5 and V5-tag antibodies. To certify the localization of V5-tagged Prx5 to the cytosol and mitochondria, mitochondrial fractionation was performed. Our results showed that CytPrx5 and MtPrx5 were successfully expressed (Figure 2(A,B)). Subsequently, we examined whether CytPrx5 and MtPrx5 are specifically localized to the cytosol and mitochondria, respectively. After performing immunohistochemistry using V5-tag antibody, the cells were stained with DAPI or MitoTracker and observed by confocal microscopy. As shown in Figure 2(C,D), we observed that CytPrx5 localized to cytosol and MtPrx5 localized to mitochondria.

### 3.3. Effects of CytPrx5/MtPrx5 on glutamate-induced ROS and mitochondrial fission

**Figure 3.**
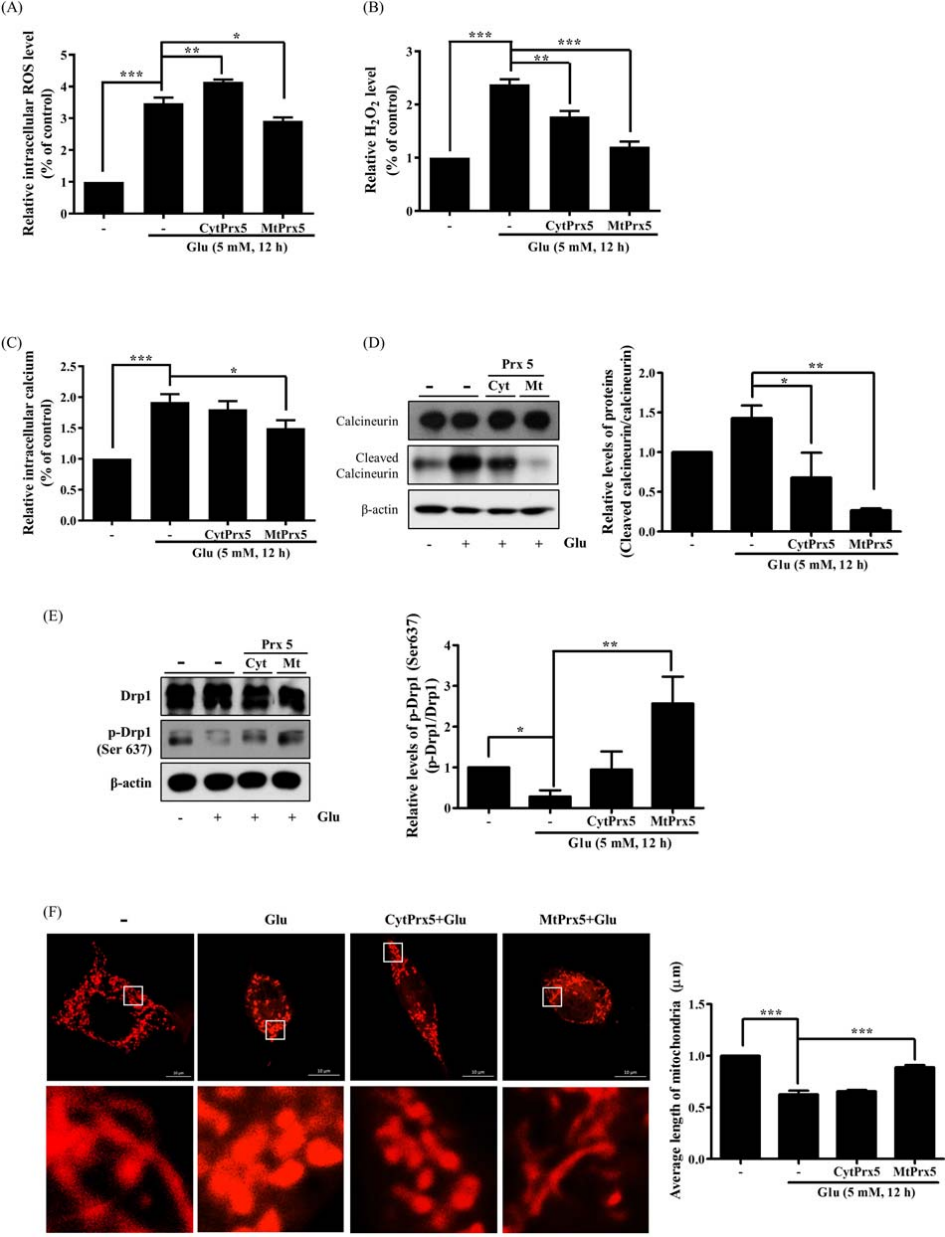
Effect of cytosolic/mitochondrial Prx5 in glutamate-induced mitochondrial dysfunction. Three types of cells (HT22, HT22-CytPrx5, and HT22-MtPrx5) were treated with 5 mM glutamate for 12 h. (A) Intracellular ROS (detected with 2.5 μM CM-H_2_DCF-DA) level was analyzed by flow cytometry. We incubated HT22 cells with fluorescent dye for 20 min. (B) Intracellular H_2_O_2_ level was measured by Fluorimetric Hydrogen Peroxide Assay Kit. (C) Levels of intracellular Ca^2+^ were detected with 2.5 μM Fluo-4 AM. We incubated HT22 cells with fluorescent dye for 20 min. (D) Levels of calcineurin and cleaved calcineurin were assessed by western blotting. (E) Changes in mitochondrial dynamics were characterized by assessing the levels of Drp1 and p-Drp1 (Ser637) by western blotting. (F) Changes in mitochondrial morphology after glutamate treatment were observed by confocal microscopy. The cells were stained with 1 μM MitoTracker (red) fluorescent dye. Images in the second row correspond to higher magnifications of the areas within white squares in the images in the first row. The histogram below the images shows the quantification of the average mitochondrial length. Mitochondrial lengths were analyzed based on individual mitochondria. Glutamate treated HT22 cells were used as a control. Values represent the mean ± SD (*n* = 4). *P < 0.05; **P < 0.01; ***P < 0.001 vs. control.

Next, we evaluated ROS levels to determine whether CytPrx5 or MtPrx5 effectively controlled glutamate-induced ROS generation by using CM-H_2_DCF-DA. Our results showed that the elevated levels of intracellular ROS induced by glutamate were reduced in both HT22-CytPrx5 and -MtPrx5 cells. Contrary to our expectations, we observed that MtPrx5 decreased the intracellular ROS level more effectively than CytPrx5 (Figure 3(A)). The analysis targeted to H_2_O_2_ level also showed a similar tendency (Figure 3(B)). Interestingly, although CytPrx5 was expressed in the cytosol which is a wide area, it failed to block intracellular ROS generation compared to MtPrx5. These results imply that mitochondrial ROS may influence cytosolic ROS generation in glutamate toxicity.

Glutamate-induced cell death is closely related to mitochondrial fission. Drp1 (dynamin-related protein 1) participates in mitochondrial fission through its phosphorylation/dephosphorylation [20]; specifically, the dephosphorylation of the 637th serine residue of Drp1 by Ca^2+^-dependent calcineurin activation leads to mitochondrial fission. Calcineurin activation is initiated by intracellular calcium accumulation and cleavage of CnA [21]. Therefore, we assessed the impact of CytPrx5 and MtPrx5 on the level of intracellular calcium and cleavage of CnA. The glutamate-induced increase in the level of intracellular calcium and cleaved CnA was more markedly reduced by MtPrx5 than CytPrx5 (Figure 3(C,D)). We then measured the effect of CytPrx5 and MtPrx5 on the glutamate-induced dephosphorylation of p-Drp1 (Ser637). As shown in Figure 3(E), MtPrx5 markedly restored the level of p-Drp1 (Ser637) after glutamate treatment. Next, to investigate any changes in mitochondrial morphology, we stained HT22, HT22-CytPrx5, and HT22-MtPrx5 cells with MitoTracker. Consistent with western blotting results, MtPrx5 maintained mitochondrial morphology and therefore protected cells against glutamate-induced mitochondrial fission. Unlike HT22-MtPrx5 cells, however, HT22-CytPrx5 cells displayed a more fragmented mitochondrial morphology (Figure 3(F)). Our results indicate that it is likely that the origin of glutamate-induced ROS overproduction is closely connected with mitochondria rather than cytosol.

### 3.4. Protective effects of CytPrx5/MtPrx5 against glutamate-induced apoptosis

**Figure 4.**
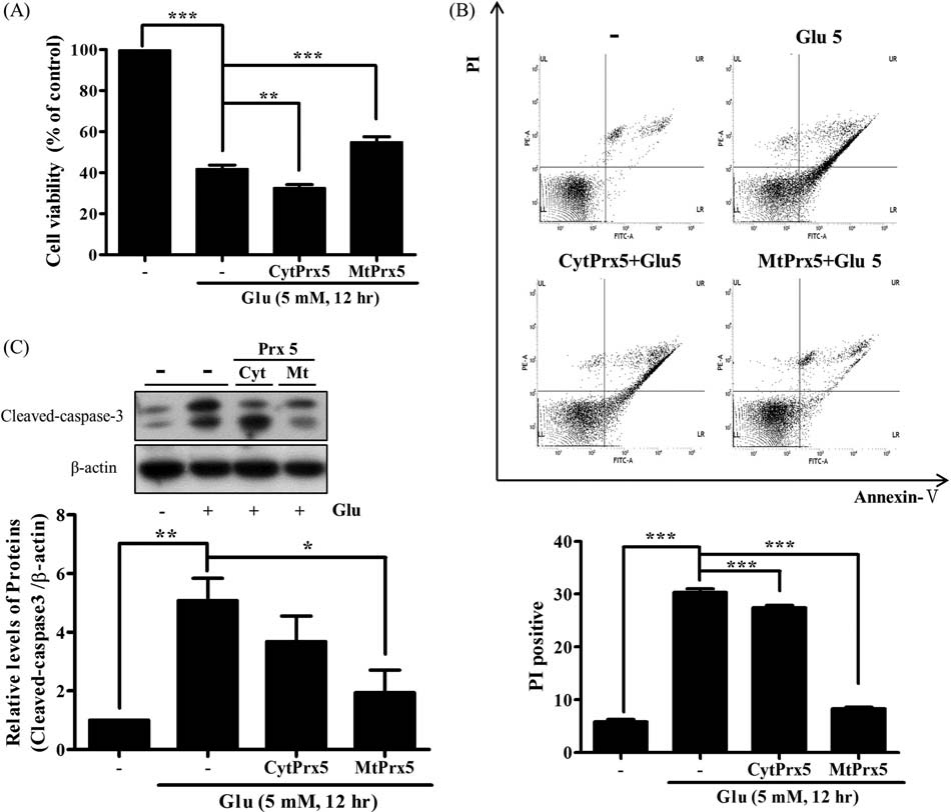
Effect of cytosolic/mitochondrial Prx5 in glutamate-induced apoptosis. Three types of cells (HT22, HT22-CytPrx5, and HT22-MtPrx5) were treated with 5 mM glutamate for 12 h. (A) Cell viability was assessed by MTT assay. (B) Analysis of cell apoptosis by flow cytometry using Annexin V-FITC/Propidium Iodide (PI) Apoptosis Detection Kit; derived histograms showing the percentage of apoptotic cells calculated by densitometry. Viable cells exhibit AnnV-/PI-; Dead cells exhibit PI+. (C) Expression level of cleaved caspase-3 was measured by western blotting. Glutamate treated HT22 cells were used as a control. Values represent the mean ± SD (*n* = 4). *P < 0.05; **P < 0.01; ***P < 0.001 vs. control.

Finally, to investigate whether CytPrx5 or MtPrx5 provides better protection against glutamate toxicity, we confirmed glutamate-induced apoptotic cell death. The cell viability of glutamate-treated HT22-MtPrx5 cells was recovered to a control level (Figure 4(A)). Subsequently, annexin V/PI double staining was performed and stained cells were analyzed by flow cytometry. The number of apoptotic HT22-MtPrx5 cells was significantly less than the number of apoptotic glutamate-treated control cells (Figure 4(B)). Therefore, MtPrx5 effectively prevented the cleavage of caspase-3, which is a representative apoptosis marker (Figure 4(C)). In comparison with MtPrx5, CytPrx5 did not sufficiently protect neuronal cells against glutamate toxicity. Overall, these results show that mitochondrial ROS is an immediate cause of glutamate-induced apoptosis.

## 4. Discussion

ROS are known to be generated by mitochondria or non-mitochondrial dependent ROS-generating enzymes, such as NADPH oxidase (NOX), xanthine oxidase, cytochrome P450, and flavin oxidases. Among all of the above, the mitochondrial respiratory chain and NOX are the major sources of ROS [22,23]. Under healthy conditions, ROS derived from each source maintain redox balance and affect cellular signaling. However, when antioxidant defenses are disrupted, ROS overproduction leads to the accumulation of excessive ROS, resulting in severe cell damage [24]. Although the precise etiology of neurodegenerative diseases has not been completely elucidated, glutamate-induced ROS overproduction is considered to be a cause of neurodegenerative disease. One consequence of ROS generation is the initiation of excitotoxicity, leading to a cascade of neuronal cell death [25]. Therefore, the regulation of ROS level can be considered a therapeutic approach to neurodegenerative diseases. To improve this approach, it is vital to establish the exact pattern of ROS generation in subcellular organelles.

In the present study, we analyzed patterns of ROS generation in glutamate toxicity using HyPer indicator, which specifically detects subcellular organelle-specific H_2_O_2_ generation [26]. Our results indicated that the levels of both cytosolic and mitochondrial H_2_O_2_ show similar time-dependent increases after glutamate treatment, and that this pattern was maintained until the end point of apoptosis. Notably, however, the level of mitochondrial H_2_O_2_ increase varied widely 12 h after treatment, unlike that of cytosolic H_2_O_2_ (Figure 1). These results indicate that mitochondrial ROS, rather than cytosolic ROS, is closely linked to glutamate-induced apoptosis. Based on these data, we hypothesized that antioxidant enzymes localized to the mitochondria play a pivotal role in neuroprotection against glutamate toxicity.

Antioxidant enzymes play a crucial role in maintaining redox balance by neutralizing ROS and other free radicals. Antioxidants can inhibit ROS generation, and further function as an up- or downstream therapeutic defense against ROS [27]. Previous studies have demonstrated that Alzheimer’s diseases patients exhibit a low level of antioxidant enzymes, such as superoxide dismutase, catalase, peroxiredoxins, etc. [28,29]. Among these enzymes, Prxs are a family of thiol-specific peroxidases. Since Prxs can catalyze the reduction of hydroperoxides and peroxynitrite, they are known to be powerful antioxidant enzymes. The Prx family can be divided into six subtypes according to subcellular localization [15,19]. Of the six Prx subtypes, the antioxidant effect of Prx5 in various cellular systems has been well established. In previous studies, we reported that Prx5 reduces LPS-induced microglia activation and protects neuronal cells from neurotoxic substances, such as β-amyloid, iron, diethylhexyl phthalate, and glutamate [13,18,30–34]. These findings highlight the necessity of further study on the relationship between the antioxidant effects and intracellular location of Prx5. Therefore, in the present study, we investigated differences in the neuroprotective effect of Prx5 according to its subcellular localization, focusing on cytosol and mitochondria.

There are two types of glutamate toxicity: receptor-dependent and receptor-independent [35,36]. Non-glutamate receptor toxicity is closely related to abnormal intracellular Ca^2+^ influx and mitochondrial dysfunction. A high level of intracellular Ca^2+^ exacerbates mitochondrial fission via calcineurin-mediated dephosphorylation of the 637th serine residue in Drp1 [20,21]. Therefore, we investigated the effect of CytPrx5 and MtPrx5 on glutamate toxicity through analysis of intracellular Ca^2+^ level and mitochondrial fission in HT22 cells (which lack glutamate receptors). Our results indicate that MtPrx5 markedly inhibits excessive Ca^2+^ influx, leading to inhibition of the cleavage of calcineurin in glutamate-treated HT22 cells. Furthermore, we showed that MtPrx5 reduced mitochondrial fission by upregulating the level of p-Drp1 (Ser637) (Figure 3). Consequently, MtPrx5 reduced apoptotic cell death (Figure 4).

In comparison, CytPrx5 showed a relatively weak protective effect against glutamate toxicity. Contrary to our expectations, even though the expression level of CytPrx5 increased more than that of MtPrx5 (Figure 2), MtPrx5 showed a stronger protective effect than CytPrx5. These findings imply that the protective effect of Prx5 is influenced by the subcellular localization, supporting the hypothesis that the location of expression makes a large difference to ROS scavenging ability, regardless of the amount or overall area of expression. The results of analysis of intracellular ROS level by CM-H_2_DCF-DA were also consistent with this hypothesis (Figure 3). Remarkably, MtPrx5 reduced intracellular ROS effectively than CytPrx5. Overall, these data reveal that the high level of cytosolic ROS in CytPrx5-HT22 cells is likely due to uncontrolled mitochondrial ROS generation.

From these result, we estimated that mitochondrial ROS might affect cytosolic ROS generation and be directly connected to glutamate-induced cell death. And, it can be inferred that the controlling mitochondrial ROS and mitochondrial function is an efficient way to reduce neuronal cell death due to glutamate toxicity. However, more precise involvement of mitochondrial ROS in neuronal cell death will be elucidated in further study. This present study suggests the need for confirmation of change in the ROS level and mitochondrial function in early stage of apoptosis (especially 3 h), not late stage. Furthermore, to clarify the association, only mitochondrial-derived ROS needs to be specifically measured. Also, in the further study, we anticipate that using the HyPer probes in the Prx5 overexpressing cells can offer more clear information about the aspect of intracellular H_2_O_2_ concentration change.

In conclusion, our data proposes that mitochondrial ROS can act as a fatal cause and direct point of glutamate-induced neurotoxic cascades, rather than cytosolic ROS. Collectively, the findings of the current study highlight the origin of ROS caused by glutamate toxicity and provide information about the causal relationship between cytosolic and mitochondrial ROS in glutamate-induced cell neuronal cell death. Furthermore, this is the first demonstration of differences in the antioxidant activity of Prx5 localized to specific subcellular compartments, including the cytosol and mitochondria. It is likely that subcellular localization determines the activity of Prx5. These findings suggest the need for further study about the enzymatic activities of Prx5 according to its expressed subcellular location and the expression level. Our results suggest that the control of mitochondrial ROS by MtPrx5 could be applied in interventions for neurodegenerative disease. We believe that the present study could also serve as a basis for further research focused on intracellular organelles.

## Author contributions

Mi Hye Kim performed the experiments and wrote the paper. Da Yeon Kim, Hong Jun Lee, Young-Ho Park, Jae-Won Huh and Dong-Seok Lee designed the study and experiments.

## Abbreviations

**Abbreviations:** ROS: reactive oxygen species; mtROS: mitochondrial ROS; PBS: phosphate-buffered saline; CM-H_2_DCF-DA: 2′,7′-dichlorofluorescein diacetate; Prx: Peroxiredoxin; CytPrx5: cytosolic Prx5; MtPrx5: mitochondrial Prx5; MTS: mitochondrial targeting sequence; Drp1: dynamin-related protein 1; FBS: fetal bovine serum
